# Estimation of Intake of Fat, Saturated Fatty Acids, and Trans Fatty Acids from Sweet and Salty Snacks Among Children and Adolescents

**DOI:** 10.3390/nu17091572

**Published:** 2025-05-02

**Authors:** Ewa Malczyk, Katarzyna Weronika Walkiewicz, Małgorzata Muc-Wierzgoń, Sylwia Dzięgielewska-Gęsiak

**Affiliations:** 1 Department of Health Sciences and Physical Education, University of Applied Sciences in Nysa, 48-300 Nysa, Poland; ewa.malczyk.nysa@gmail.com; 2 Department of Internal Diseases Propaedeutics and Emergency Medicine, Faculty of Public Health in Bytom, Medical University of Silesia in Katowice, Piekarska 18, 44-902 Bytom, Poland; kk.walkiewicz@gmail.com (K.W.W.); sgesiak@sum.edu.pl (S.D.-G.)

**Keywords:** children and adolescents, snack consumption, dietary fat, saturated fatty acids, trans fatty acids, dietary habits

## Abstract

**Objectives**: The study aimed to assess the intake of total fat, saturated fatty acids (SFAs), and trans fatty acids (TFAs) from sweet and salty snacks among Polish schoolchildren and to evaluate differences by age, gender, and nutritional status. **Methods**: A cross-sectional study was conducted among 362 pupils aged 10–15 years. Snack consumption was assessed using a validated food frequency questionnaire. Fat content was estimated based on product labels and databases, and the Estimated Daily Intake (EDI) of fats was calculated. Intake levels were compared to dietary recommendations and analyzed by gender, age, and BMI category. **Results**: The mean fat intake from snacks was 34.5 g/day, covering nearly 47% of the recommended daily intake. Over 12% of students exceeded total fat recommendations, 20% exceeded SFA limits, and more than 30% surpassed the TFA intake threshold. The highest intake of fats was observed among 10-year-olds. Over 60% of students in this age group exceeded the TFA’s upper intake level. Girls showed slightly higher intakes of total fat and SFAs than boys, although the differences were not statistically significant. Fat intake was highest among children with overweight or obesity. **Conclusions**: Snacks are a major contributor to unhealthy fat intake among school-aged children, particularly in the youngest age group and those with excess body weight. Early, targeted nutrition education and regulatory strategies are needed to promote healthier snacking habits and reduce the long-term risk of diet-related non-communicable diseases.

## 1. Introduction

Snacks are a simple and quick way to satisfy so-called first hunger. They are commonly considered the most convenient alternative to regular food when you do not have time to eat or as a supplement between regular meals. There are many definitions of a snack. Generally, a snack is a small portion of food eaten between regular meals. Snacks can be sweet, savory, or healthy, and they serve various purposes, such as satisfying hunger, providing energy, or indulging in a treat [1]. According to a Nielsen IQ report, from April 2023 to March 2024, the snack market in Poland reached PLN 27.1 billion (6.77 trillion USD), with sweet snacks dominating. Among sweet snacks, Poles most often bought pre-packaged cakes and cookies (3.9–0.97 billion USD), pralines (2.9 billion USD), and chocolate bars (2.8 billion USD); among salty snacks, chips and crisps dominated (5 billion USD) [2].

In Poland, sweets are a regular part of the diet for most consumers (91%) [3]. In 2024, the European market for confectionery (this segment includes chocolate products, confectionery, and preserved pastry products) and snacks was valued at approximately 256.20 billion USD, with a projected annual growth rate (CAGR) of 5.18% between 2024 and 2029 [3]. On average, Poles consume between 5.7 and 5.9 kg of chocolate per person per year [4], which is less than in leading European countries. In contrast, several other European countries have higher per capita chocolate consumption [5,6], such as Switzerland: 8.8 kg, Austria: 8.1 kg, and Germany: 7.9 kg.

Sweet and salty snacks mainly provide simple sugars and fats. In European Union (EU) legislation, fat is defined as total fat, including phospholipids. The basis for this definition is how total fat is extracted from food products. In this context, the term “fat” refers to all lipid fractions, both free and bound, in plant and animal tissues, extracted together from a food sample using generally accepted analytical methods [7].

Dietary fats play a key role in health by providing energy, promoting cell growth, developing during early life, and aiding in the absorption of fat-soluble vitamins (A, D, E, and K). They also contribute to hormone production (steroid hormones, prostaglandins) and brain function—omega-3 and omega-6 fatty acids are crucial for cognitive function, mood regulation, and neuroprotection [8]. However, not all fats are created equal in terms of health effects [9]. Types of dietary fats include monounsaturated fats (MUFA), polyunsaturated fats (PUFA), saturated fats (SFAs), and trans fats (TFAs) [10]. Metabolic and epidemiological studies have shown that consumption of saturated and trans fats accelerates cell apoptosis, elevates inflammatory markers (such as C-reactive protein, fibrinogen, interleukin 6, ICAM-1, VCAM-1, PGE, TNF alpha), promotes insulin resistance, promotes weight gain, contributes to visceral fat accumulation, and contributes to liver dysfunction. This results in an increased risk of various chronic diseases, including cardiovascular disease (CVD), cancer (especially breast, prostate, and colon), obesity, type 2 diabetes, and non-alcoholic fatty liver disease [11,12,13,14,15,16]. Childhood obesity is also linked to increased risk of depression and anxiety, lower self-esteem, and poorer academic performance.

There is a correlation between saturated fat intake and elevated levels of triglycerides, low-density lipoprotein (LDL) cholesterol (as well as reduced levels of high-density lipoprotein cholesterol (HDL-C) [17]. A Cochrane review found that reducing saturated fat intake for at least two years led to a reduction in cardiovascular events, although it had no significant effect on total mortality or cardiovascular mortality [18].

Trans isomers of fatty acids (so-called trans fats) are classified according to the two main sources from which they are derived: natural so-called r-TFAs (ruminant trans fatty acids) and industrially produced so-called i-TFAs (industrially produced trans fatty acids). i-TFAs are primarily formed by partial hydrogenation of vegetable or fish oils as an unfavorable side effect of industrial hardening processes [7]. Trans fats raise inflammatory markers and low-density lipoprotein cholesterol, while lowering high-density lipoprotein (HDL) cholesterol, leading to an unfavorable lipid profile. For example, a meta-analysis showed that a 2% increase in energy intake from trans fats is associated with a 23% increase in cardiovascular risk [19]. A 30% increase in the risk of developing CHD and an 18% increase in the risk of death were reported for high intake of i-TFAs alone.

The TFAs have negative effects on fertility in both men and women. In women, elevated intake of TFAs has been linked to the most negative effects on ovulation, pregnancy length, fetal malformations, and increased risk of fetal loss [20,21]. In men, higher TFA intake is inversely associated with sperm concentration and total sperm count. In addition, there is a positive correlation between trans fatty acid intake and asthenospermia [21].

The lack of adequate research on TFA intake in children may be due to the fact that most epidemiological studies on TFA intake have evaluated cardiovascular effects, which typically do not occur until later in life [22].

Saturated fatty acids and trans fatty acids are a constant ingredient in processed snacks, as they affect their taste, texture, and shelf life. Although trans fats are increasingly being eliminated due to international and national regulations, they can still be present in some products. The 2023 National Nutrition Test of Poles indicates that sweets—such as candies, cakes, cookies, candy bars, yeast cakes, and waffles—are chosen by 44% of respondents [23]. According to the same survey, young adults (18–24 years old) were the most likely to consume sweet snacks (63%) as well as yogurt, cheese, and dairy desserts (42%).

The World Health Organization (WHO) recommends that in individuals aged 2 years and older, saturated fatty acids should constitute no more than 10% of total daily energy intake (EI), while trans fatty acids should contribute less than 1% of total daily EI [24]. According to the Nutrition Standards for the Polish Population, the intake levels of saturated fatty acids and trans fatty acid isomers across all age groups should be minimized as much as possible while ensuring the nutritional adequacy of the diet. Moreover, the total fat intake for children and adolescents aged 3–18 years should provide 30–40% of total energy intake [7].

The aim of this study was to assess the snack consumption behaviors of Polish adolescents aged 10–15 years, and to estimate their intake of total fat, saturated fatty acids (SFAs), and trans fatty acid isomers (TFAs) from sweet and salty snacks.

## 2. Materials and Methods

### 2.1. Materials

#### 2.1.1. Ethics

The study protocol was reviewed by the Bioethics Committee of the Silesian Medical University in Katowice, which concluded that it did not meet the criteria of a medical experiment and did not require ethical approval. Nonetheless, in accordance with the 2013 Declaration of Helsinki, participation was voluntary and based on informed consent. Participants received detailed study information during meetings with school administrators. Additionally, letters outlining the project’s purpose and methodology were sent to parents or guardians. Written consent was obtained from school principals, parents or guardians, and students aged 16 or older. To protect privacy, data were pseudonymized, making individual identification possible only with an additional key.

#### 2.1.2. Participants

A total of 362 students aged 10–15 years, all attending public schools in Poland, were included in the study. Inclusion criteria were the school administration’s consent to conduct the survey and written consent from a parent.

### 2.2. Methods

#### 2.2.1. Questionnaire Development and Validation

The questionnaire was developed based on a comprehensive literature review on children’s eating behaviors and snack preferences. Its content validity was assessed by a panel of experts in pediatric nutrition and dietary assessment. To evaluate test–retest reliability, a pilot study was conducted twice among a sample of 50 schoolchildren (10 from each age group), with a 30-day interval between administrations. Internal consistency was measured using Cronbach’s alpha coefficient (0.80), and test–retest reliability was assessed using intraclass correlation coefficients (ICCs).

During the development phase, 30 products were preselected based on data from national dietary surveys and interviews with children, focusing on commonly consumed sweet and salty snacks. The final questionnaire was completed under the supervision of a trained dietitian, who was available to provide clarification during the survey.

#### 2.2.2. Questionnaire Structure

The final version of the questionnaire included:

- Demographic data: gender, age, weight, and height;
- Eating behaviors: frequency and portion sizes of 20 sweet and salty snack products.

A list of 20 products was selected for detailed analysis, including chocolate, chocolate bars, wafers, cookies, crackers, crisps, and fries. Frequency of consumption was assessed using a predefined scale:

Several times a day (1–3 times),
Several times a week (1–6 times),
Several times a month (1–3 times),
Rarely (up to 5 times a year or more than 5 times a year),
No consumption.

#### 2.2.3. Portion Size Estimation

Portion sizes were standardized using color photographs from the *Album of Photographs of Food Products and Dishes* [25], supplemented by additional images reflecting portion sizes established during the validation phase. Respondents selected the portion size most closely resembling their usual intake.

#### 2.2.4. Assessment of Snacking Preferences

Snacking preferences were assessed using a 4-point Likert scale:

1. Never
2. Rarely
3. Often
4. Very often

Participants responded separately regarding their preference for sweet and salty snacks. Higher scores indicated stronger preferences. Based on the median score within each category, preferences were dichotomized into “low” and “high” for further analysis.

#### 2.2.5. Estimation of Total Fat, Saturated Fatty Acids (SFAs), and Trans Fatty Acids (TFAs) Intake

The fat content, saturated fatty acids (SFAs), and trans fatty acids (TFAs) of individual snack products were determined based on nutritional labeling and data available on the official TFAs information website [26,27,28]. Table 1 presents examples of confectionery and salty snacks commonly consumed by children and adolescents, selected from a broader pool of products evaluated in the initial screening.

Filled wafers, chocolate bars, and coconut-based candies were particularly high in total fat and SFAs, often exceeding 30 g and 20 g per 100 g, respectively. In contrast, baked potato chips and pretzels contained substantially lower amounts of fat and saturated fat. Trans fatty acids (TFAs) were also more prevalent in shortbread cookies, glazed donuts, and coconut candy bars, while being nearly negligible in certain salty snacks such as pretzels or baked chips. These findings reflect significant variation in the nutritional profiles of snacks available to children and adolescents.

Estimated Daily Intake (EDI) of FATs, SFAs, and TFAs was calculated using the formula:

EDI = F × M × R (g/day)(1)

where F is the portion size (g), M is the content of FATs, SFAs, or TFAs per 100 g of product, and R is the reported frequency of consumption converted to a daily equivalent: several times per day: R/1; several times per week: R/7; several times per month: R/30; rarely: R/365.

Example:

If a participant consumed 20 g of milk chocolate (32.8 g fat/100 g) five times per week:

EDI = 20 × (32.8/100) × (5/7) = 4.7 g fat/day.

#### 2.2.6. Participant Classification and Reference Values

Participants were stratified by gender, age, and nutritional status. Nutritional status was determined using BMI-for-age percentile charts [29]. To assess the adequacy of fat intake, weighted average reference values for daily energy intake and recommended intake levels of FATs, SFAs, and TFAs were calculated for the study group, based on national dietary standards [7].

The percentages of recommended daily intakes of FATs, SFAs, and TFAs were then calculated for each participant and analyzed by gender.

The results are presented in Table 2.

#### 2.2.7. Statistical Analysis

Statistical analyses were performed using Statistica software, version 13.0. The normality of data distribution was assessed using the Shapiro–Wilk test.

To examine differences in fat, saturated fatty acid (SFAs), and trans fatty acid (TFAs) intake according to age, sex, and nutritional status (based on BMI), appropriate statistical tests were applied depending on data distribution: the Mann–Whitney U test and Kruskal–Wallis test were used for non-normally distributed variables, while one-way analysis of variance (ANOVA) followed by the Tukey post hoc test was used for normally distributed variables. A *p*-value of <0.05 was considered statistically significant.

## 3. Results

The study included 362 school pupils: 192 girls (53.0%) and 170 boys (47.0%). The age distribution was as follows: 50 pupils aged 10 and 12 years (13.8%), 49 aged 11 years (13.5%), 47 aged 13 years (13.0%), 82 aged 14 years (22.7%), and 84 aged 15 years (23.2%).

Based on BMI-for-age percentile charts, 253 participants (69.9%) had normal weight, 43 (11.9%) were classified as underweight, 56 (15.5%) as overweight, and 10 (2.7%) as obese (Table 3).

When stratified by sex, girls more frequently had a normal weight compared to boys (82.3% vs. 55.9%). Conversely, underweight was considerably more common among boys (24.1%) than girls (1.1%). Overweight was slightly more prevalent among boys (17.6%) than girls (13.5%), while obesity affected 3.1% of girls and 2.4% of boys (Table 4 and Table 5).

Age-specific patterns also varied between sexes. Among girls, all cases of obesity occurred exclusively in the 15-year-old group. In contrast, boys with obesity were mostly observed in the youngest group (10 years). Overweight was most prevalent in 15-year-old girls (57.7%) and 12-year-old boys (30.0%)—Table 4 and Table 5. Interestingly, underweight boys were present in nearly every age group, with the highest percentages noted among those aged 13–15 years.

### 3.1. Estimation of Fat Intake

The mean daily fat intake from confectionery and salty snacks among the entire study group was 34.5 g/day, representing 46.8% of the recommended daily fat intake. The values ranged widely, from as little as 0.76 g/day to as much as 117.6 g/day. Girls consumed slightly more fat than boys on average (36.5 g/day vs. 32.2 g/day), corresponding to 52.6% and 41.0% of their respective dietary fat requirements. However, this difference was not statistically significant (Table 6).

A significant age-related effect on fat intake was observed (*p* < 0.05). The highest intake was recorded among 10-year-olds (41.4 g/day, 65.3% of the reference intake), with a noticeable drop in the 11- and 12-year-old groups (25.0 and 29.7 g/day, respectively). In older adolescents (13–15 years), fat intake stabilized between 33.3 and 38.7 g/day. Among girls, fat intake was highest in 14-year-olds (46.3 g/day, covering 63.8% of the recommended intake) and lowest in the 13-year-olds (29.4 g/day, 41.8%). Among boys, the greatest intake was noted in the 10-year-olds (42.5 g/day), with markedly lower values observed in 11-year-olds (21.7 g/day) and a gradual increase thereafter (Table 7 and Table 8).

With regard to nutritional status, there was a trend of increasing fat intake with increasing BMI category. Underweight participants consumed an average of 30.3 g/day, compared to 38.5 g/day among those who were overweight and 41.6 g/day among those with obesity. While the overall effect of BMI on fat intake was not statistically significant, selected subgroups showed marked differences. For instance, boys with overweight or obesity at age 10 consumed substantially more fat (73.1 g/day and 50.7 g/day, respectively) than their normal-weight peers (30.6 g/day). Similar patterns were observed at age 13, where boys with overweight reported the highest relative fat intake (118.5% of the recommendation).

Among girls, the highest intake relative to dietary requirements was observed in 11-year-olds with overweight (51.3 g/day, 80.4% of recommendation), while the lowest was in 13-year-olds with underweight (18.8 g/day, 26.7%). Notably, no data were available for underweight or obese girls in most age categories, likely due to low group numbers.

### 3.2. SFAs Intake Estimation

The average intake of saturated fatty acids (SFAs) from confectionery and salty snacks among the study participants was 15.3 g/day, which corresponds to 59.1% of the recommended daily maximum. Girls consumed more SFAs than boys (16.1 g vs. 14.4 g/day), covering 66.0% and 52.2% of their respective daily limits. However, the differences between sexes were not statistically significant (Table 9).

Statistically significant differences in SFA intake were observed across age groups (*p* < 0.05). The highest average intake was recorded among 10-year-olds (18.9 g/day, 84.8% of the recommendation), whereas the lowest was found in 11-year-olds (10.8 g/day, 46.6%). From age 12 onwards, intake levels increased gradually, ranging from 12.7 g to 17.2 g/day, corresponding to 51–63% of the allowable daily intake.

With regard to nutritional status, overweight and obese students had the highest SFA consumption (18.0 g and 18.3 g/day, respectively), corresponding to 70.6% and 73.8% of the maximum daily limit. In contrast, underweight individuals consumed the least (13.8 g/day; 48.6% of the daily limit) and those with normal weight averaged 14.8 g/day (58.0%). This trend was observed in both sexes.

Among girls, the highest SFA intake was noted in 10-year-olds with normal weight (19.7 g/day; 92.5% of the limit), and in 11-year-olds with overweight (20.4 g/day; 91.1%)—Table 10.

In boys, the highest values were observed in 10-year-olds with overweight (36.2 g/day; 157.4% of the limit) and in 13-year-olds with overweight (40.6 g/day; 145.5%). Notably, some boys with overweight or obesity in younger age groups substantially exceeded the recommended maximum intake (Table 11).

Overweight and obese individuals had the highest intake of saturated fatty acids. The average intake of SFA in these individuals ranged from 18.0 to 18.3 g, which was more than 70% of the daily intake limit for this dietary component. Normal-weight students averaged an intake of 14.8 g of SFA (58% of the daily intake limit).

Although not statistically significant for BMI overall, these subgroup differences suggest that higher fat mass may be associated with excessive consumption of SFAs from snacks. Furthermore, wide interindividual variation within BMI and age categories highlights the heterogeneity of dietary habits in this population.

### 3.3. TFAs Intake Estimation

The mean daily intake of trans fatty acid isomers among the surveyed students was 2.5 g/day, corresponding to 96.2% of the recommended daily limit. Girls showed a slightly higher intake than boys (2.5 g/day vs. 2.4 g/day), resulting in 104.2% and 85.7% of their respective dietary limits. However, these differences were not statistically significant (Table 12).

Statistically significant differences were observed across age groups (*p* < 0.05). The highest average TFA intake was recorded among 10-year-olds (3.6 g/day, 163.6% of the recommended intake), far exceeding the suggested maximum. The lowest intake was observed in 11- and 12-year-olds (1.5 g/day and 1.8 g/day, respectively), while adolescents aged 13–15 consumed approximately 2.2–2.8 g/day, close to or slightly exceeding the allowable limit (Table 11).

Age significantly affected TFA intake (*p* < 0.05). The highest average intake was recorded among 10-year-olds (3.6 g/day; 163.6% of the recommendation). Girls in this age group consumed particularly high amounts, with 10-year-old girls with overweight recording an average intake of 3.9 g/day—185.7% of the recommended maximum—Table 13.

In contrast, the lowest intake was observed among 11- and 12-year-olds (1.5 and 1.8 g/day, respectively), both in girls and boys—Table 14.

Among girls, TFA intake exceeded recommendations in several age and BMI subgroups. For example, 14- and 15-year-old girls exceeded the threshold on average (108.0% and 107.7%, respectively), with overweight girls aged 15 showing a particularly high intake of 3.7 g/day (142.0%). Interestingly, in most age groups, girls with overweight consumed more TFAs than their normal-weight peers.

Among boys, extremely high TFA intake was noted in certain individuals. Overweight boys aged 13 consumed an average of 12.8 g/day (457.1% of the limit), while obese 10-year-old boys reached 3.9 g/day (169.5%). Nevertheless, not all boys exceeded the intake limit: boys aged 11 and 12 had average intakes below 2 g/day—Table 14.

In total, 115 students (31.8%) exceeded the recommended TFA intake. The proportion was similar between girls (30.7%) and boys (32.9%). However, breakdown by age showed that excessiveness was most common among 10-year-olds (64.0%), followed by overweight (42.8%) and obese (40.0%) participants.

These findings highlight that both girls and boys, particularly those with excess body weight and aged 10–15, are at elevated risk of excessive TFA intake.

Across the entire study population, 12.2% of participants exceeded the recommended daily intake of total fat from confectionery and salty snacks. In contrast, exceedance was more common for saturated fatty acids (SFAs) and trans fatty acid isomers (TFAs), reported in 19.9% and 31.8% of students, respectively (Table 15).

Girls were more likely to exceed the recommendations for total fat and SFAs than boys (14.6% vs. 9.4% for total fat; 23.4% vs. 15.9% for SFAs). In contrast, boys slightly more frequently exceeded TFA limits (32.9% vs. 30.7%).

**Figure 1:**
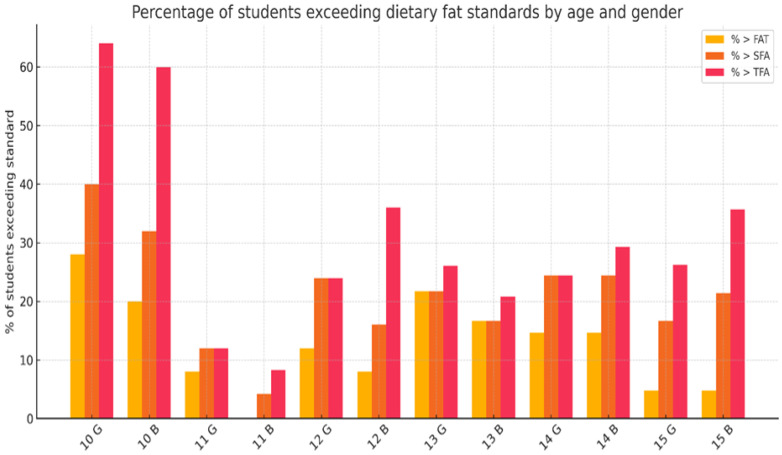
Percentage of students exceeding dietary intake recommendations for total fat (FATs), saturated fatty acids (SFAs), and trans fatty acids (TFAs) from snacks, by age and gender. G—girls, B—boys.

Age was a major determinant of dietary fat excess. Among 10-year-olds, nearly two-thirds (64.0%) surpassed the TFA intake recommendation, while 26.0% exceeded SFA limits and 24.0% exceeded total fat intake. The lowest proportions of exceedance were observed among 11-year-olds (4.1% for total fat; 8.2% for SFAs; 14.3% for TFAs)—Figure 1.

When stratified by nutritional status, overweight students most frequently exceeded recommendations: 21.4% for total fat, 30.4% for SFAs, and 42.8% for TFAs. Obese students also showed high rates of excess (40.0% for TFAs), although their numbers were small. In contrast, underweight and normal-weight students were less likely to exceed intake thresholds across all types of fat.

Figure 1 illustrates the proportion of students exceeding dietary recommendations for total fat, SFAs, and TFAs intake, disaggregated by age and gender. The highest exceedance rates were recorded among 10-year-olds, particularly for TFAs, where more than 60% of both girls and boys surpassed the recommended limits. Among older students, the percentage of exceedance was generally lower but remained notable, especially in 14- and 15-year-olds. Girls tended to exceed the fat and SFA recommendations more often in early adolescence, while boys exceeded TFA limits more frequently, particularly at ages 10 and 15.

## 4. Discussion

The body’s fat requirements under homeostatic conditions depend on various factors such as age, sex, type of physical activity, and physiological status (e.g., pregnancy, lactation, menopause, and andropause) [7]. According to the latest World Health Organization dietary recommendations for the prevention of unhealthy weight gain, fat intake should be limited to 30% of total energy intake [30]. In the prevention of diet-related diseases—as well as for proper development and the maintenance of health—the quality of fat consumed is more important than the total amount. In particular, efforts should be made to eliminate industrially produced trans fats and animal fats rich in saturated fatty acids [31].

Given the widespread availability and marketing of high-fat, ultra-processed snacks targeted at children and adolescents, it is important to evaluate the nutritional quality of these products [32,33,34]. Based on earlier reports indicating the popularity of sweet and salty snacks among Polish youth, a photographic food album with 20 snack items was used. From this list, five of the most frequently selected categories—including chocolate bars, wafers, crisps, and cookies—were chosen in collaboration with study participants to reflect their real-life preferences. Nutritional data for these snacks are presented in Table 1 and serve as the basis for estimating daily fat intake.

Our findings are consistent with broader trends reported by Beltrá et al. [32], who analyzed 3209 food products from the BADALI Food Database in Spain. Among all products intended for children or adolescents, 61.5% were high in fat, 58.5% in free sugars, 45.4% in saturated fat, and 45% in sodium. Snacks contribute approximately 25.0% of adolescents’ daily total fat intake and 25.4% of their saturated fat intake [33]. In the study by Larson et al. [34], adolescents consumed an average of 2.2 daily servings of energy-dense snacks and 4.3 snacks per day, with many consumed outside the home. Findings from the study by Kotowska et al. [35] indicate that 13.4% of young Polish individuals consumed sweets between main meals on a daily basis, 37.7% did so several times per week, and 16.2% once per week. Chips, crackers, and puffed snacks were also reported as frequent snacks, with daily consumption observed in 2.1% of respondents, several times per week in 13.4%, and once per week in 21.4%.

This is particularly concerning given the growing body of evidence suggesting that dietary habits are established early in life, tend to persist from childhood, and have long-term consequences for future health outcomes [36,37,38,39].

More than 12% of participants exceeded the recommended fat intake based on snack consumption alone, particularly among girls, 10-year-olds, and individuals with overweight. Girls exhibited a slightly higher fat intake than boys (~4 g/day). This may reflect gender-based differences in snacking behavior, emotional eating, or early body image awareness, particularly among preadolescent girls. While sex differences were not statistically significant, a visible trend was observed, consistent with earlier reports [39]. This partially aligns with the study by Hoy et al. [40], which found that snack consumption is higher among children aged 6–11 years compared to adolescents aged 12–19 years. Younger children may rely more on habitual household snacking patterns, while older youth may be more influenced by peer norms, diet culture, or body image concerns [41,42].

The mean saturated fatty acid (SFA) intake from snacks was 15.3 g/day, covering 59% of the maximum recommended daily intake. Girls consumed slightly more SFAs (16.1 g/day) than boys (14.4 g/day). The highest intake was observed among 10-year-olds (18.9 g/day), significantly higher than that of 11-year-olds (10.8 g/day; *p* < 0.05). This suggests a steep drop in SFA intake with age.

One methodological consideration is that nutrient intake estimations in our study were based on chronological age and sex categories. However, 10-year-old children represent a highly heterogeneous group in terms of biological development—some may already be entering puberty, while others remain in early childhood stages. This variability can influence both physiological needs and food preferences. Therefore, interpreting the unexpectedly high intake of saturated and trans fats in this group requires caution. Future research should consider using continuous variables such as BMI-for-age z-scores or biological maturation indicators to better reflect individual differences in growth and metabolic demands.

Notably, children with overweight or obesity showed the highest mean SFA intakes (18.0–18.3 g/day), exceeding the recommendations. This pattern reinforces the link between excess body weight and increased intake of energy-dense snacks, suggesting that BMI may be a more informative marker than age alone in dietary risk assessment. Trends in total fat intake and changes in fatty acid intake among Polish youths aged 11–15 years over a 24-year period were analyzed by Charzewska et al. [42]. The lowest percentage of energy from fat (EF%) was observed in 1999/2000—34.4% in boys and 32.7% in girls. However, in the most recent study period (2005/2006), an increase was noted—up to 35.1% in boys and 33.7% in girls.

Similar trends were observed in the Korea National Health and Nutrition Examination Survey (KNHANES VI-1-2013) [43]. Based on 24 h recall data collected from 6406 adolescents between 2007 and 2017, total fat intake increased from 54.3 g (21.7% of energy) to 61.8 g (25.2% of energy). Intakes of saturated fatty acids (SFAs) and monounsaturated fatty acids (MUFAs) also rose—from 17.8 g (7.1% of energy) and 17.2 g (6.8% of energy) to 20.6 g (8.4%) and 20.7 g (8.4%), respectively. The proportion of Korean adolescents consuming more than 30% of total energy from fat increased significantly from 13.7% to 27.5%, with all gender and age groups showing upward trends. Additionally, the percentage of individuals obtaining more than 8% of energy from SFAs rose from 36.0% in 2007–2009 to 49.7% in 2016–2017 [44].

In contrast, results from the HELENA (Healthy Lifestyle in Europe by Nutrition in Adolescence) Study showed that cholesterol and total fat intakes among 1590 European adolescents aged 12.5–17.5 years were generally in line with recommendations in both sexes; however, SFA intake exceeded the recommendations by approximately 40% [45]. Similarly, among adolescents aged 15–18 years living in Gdynia (Poland), SFA intake was within the recommended range of 45.5% of respondents, moderately high in 12%, and very high in 5.8% [46]. Importantly, high SFA intake in youth has been associated with greater adiposity and increased cardiometabolic risk [36,37,38].

Trans fatty acid (TFA) intake averaged 2.4 g/day, corresponding to more than 90% of the WHO-recommended limit of <1% of total daily energy intake. The highest values were observed in 10-year-olds (3.6 g/day) and in participants with overweight or obesity (2.9–3.2 g/day). These amounts exceed the acceptable intake level of 2.6 g/day and highlight the continued dietary relevance of TFAs despite national efforts to eliminate them. In 2018, the World Health Organization launched the REPLACE initiative to eliminate industrial TFAs. Poland was among the first countries to receive WHO certification for trans fat elimination. However, our findings show that ultra-processed snacks remain a dietary source of TFAs for school-aged children [47].

It is important to note that our estimates reflect only fat intake from selected snacks and do not capture other dietary sources, which may lead to underestimation of total fat and fatty acid consumption.

Overall, children with overweight or obesity had the highest intakes of total fat, SFAs, and TFAs, which may contribute to excess body weight and long-term metabolic risk. While sex and BMI did not reach statistical significance, visible trends indicate that these groups are more likely to exceed intake recommendations. These results support earlier research linking snack consumption with adiposity in youth [47] and emphasize the importance of early dietary interventions.

Taken together, our findings underscore the need for targeted nutrition education for children and parents to promote healthier snacking habits early in life. Regulatory efforts to limit the marketing and availability of high-fat snacks, particularly those high in SFAs and TFAs, remain crucial. Interventions should begin before adolescence to prevent excessive intake patterns and reduce the risk of obesity and related chronic diseases.

### Limitations of the Study

This study has several limitations that should be considered. First, respondents were recruited from schools located in a single region of Poland, which may limit the generalizability of the results to the broader population of Polish children and adolescents. Second, although the sample size of 362 students was adequate for exploratory purposes, it may not fully capture the heterogeneity of dietary behaviors and socioeconomic backgrounds.

Importantly, data on socioeconomic status, parental education, type of school (public vs. private), and physical activity levels were not collected. These factors may influence food choices and energy requirements, and their absence restricts the depth of interpretation.

Additionally, information on pubertal development was not collected, and no clinical assessment of maturation status was performed. As a result, chronological age was used as a proxy for biological development, which may not accurately reflect individual variability in growth patterns, physiological needs, or dietary preferences, especially in early adolescence. These biological and clinical indicators will be included in our next studies.

Their absence limits the ability to fully contextualize the results and to explore potential interactions between clinical, environmental, behavioral, and nutritional determinants. Future studies should incorporate these variables to provide a more comprehensive understanding of snack-related fat intake among school-aged populations.

## 5. Conclusions

This study reveals excessive dietary fat intake among school-aged children, with over 12% exceeding recommendations for total fat, 20% for saturated fatty acids (SFAs), and over 30% for trans fatty acids (TFAs). The highest intake levels were observed among 10-year-olds, over 60% of whom surpassed the acceptable TFA intake, identifying them as a high-risk group for diet-related non-communicable diseases (NCDs).

These findings highlight the urgent need for age-specific public health interventions, including nutrition education, regulatory measures, and supportive food environments. Involving parents and caregivers is crucial for fostering healthy eating behaviors from an early age and promoting long-term health among children and adolescents.

## Figures and Tables

**Table 1:** Nutritional value of selected sweets and salty snacks.

Product Category	Fat (g/100 g)	SFA (g/100 g)	TFA (g/100 g)	Source
Filled wafers	29.0	18.0	1.2	Product label
Potato chips	33.0	11.0	0.5	IŻŻ database
Chocolate bar (milk)	35.0	21.0	1.4	Manufacturer
Puffed corn snacks	27.0	9.5	0.8	Product label
Shortbread cookies	26.5	13.5	2.1	IŻŻ database
Nut chocolate spread	31.0	10.0	0.6	Product label
Salted pretzels	5.5	1.1	0.1	IŻŻ database
Coconut candy bar	34.5	22.0	1.9	Manufacturer
Popcorn (butter flavor)	28.0	12.5	0.4	Product label
Glazed donuts	23.0	11.0	2.3	Product label

**Table 2:** Estimated average energy requirement (EAR) and recommended intake of total fat, saturated fatty acids (SFAs), and trans fatty acids (TFAs) for boys and girls, based on age and physical activity level (PAL = 1.6). Values assume 30% of total energy intake is derived from fat.

Gender	Age [yrs]	Energy Requirements [kcal/day]	Fat Intake Norm [g]	Saturated Fatty Acids Intake Norm [g]	Trans Fatty Acids Intake Norm [g]
Boys	10	1965	65.5	<23.0	<2.3
	11	2074	69.1	<24.3	<2.4
	12	2217	73.9	<25.9	<2.6
	13	2384	79.5	<27.9	<2.8
	14	2558	85.3	<29.9	<3.0
	15	2712	90.4	<31.7	<3.2
Girls	10	1824	60.8	<21.3	<2.1
	11	1914	63.8	<22.4	<2.2
	12	2016	67.4	<23.6	<2.4
	13	2111	70.4	<24.7	<2.5
	14	2179	72.6	<25.5	<2.5
	15	2220	74.0	<26.0	<2.6

**Table 3:** Characteristics of the study group—total sample regardless of sex.

Age [Years]	Total		Underweight		Normal Weight		Overweight		Obesity	
	N	%	N	%	N	%	N	%	N	%
	362	100.0	43	11.9	253	69.9	56	15.5	10	2.7
10	50	13.8	0	0.0	37	14.6	9	16.1	4	40.0
11	49	13.5	3	6.9	38	15.0	8	14.3	0	0.0
12	50	13.8	9	20.9	32	12.6	9	16.1	0	0.0
13	47	13.0	11	25.6	27	10.7	9	16.1	0	0.0
14	82	22.7	10	23.3	68	26.9	4	7.1	0	0.0
15	84	23.2	10	23.3	51	20.2	17	30.3	6	60.0

**Table 4:** Characteristics of the study group—girls.

Age [Years]	Total		Underweight		Normal Weight		Overweight		Obesity	
	N	%	N	%	N	%	N	%	N	%
	192	53.0	2	1.1	158	82.3	26	13.5	6	3.1
10	22	11.5	0	0.0	19	12.0	3	11.5	0	0.0
11	28	14.6	0	0.0	26	16.5	2	7.7	0	0.0
12	26	13.5	0	0.0	26	16.5	0	0.0	0	0.0
13	20	10.4	2	100.0	12	7.6	6	23.1	0	0.0
14	48	25.0	0	0.0	48	30.4	0	0.0	0	0.0
15	48	25.0	0	0.0	27	17.1	15	57.7	6	100.0

**Table 5:** Characteristics of the study group—boys.

Age [Years]	Total		Underweight		Normal Weight		Overweight		Obesity	
	N	%	N	%	N	%	N	%	N	%
	170	47.0	41	24.1	95	55.9	30	17.6	4	2.4
10	28	16.5	0	0.0	18	18.9	6	20.0	4	100.0
11	21	12.3	3	7.3	12	12.6	6	20.0	0	0.0
12	24	14.1	9	22.0	6	6.3	9	30.0	0	0.0
13	27	15.9	9	22.0	15	15.8	3	10.0	0	0.0
14	34	20.0	10	24.4	20	21.1	4	13.3	0	0.0
15	36	21.2	10	24.4	27	28.4	2	6.7	0	0.0

**Table 6:** The average daily fat intake by gender, age, and nutritional status, along with the percentage of dietary fat requirement coverage.

		Fat	Weighted Average Nutrition Standard	% of Standard Coverage
	Total, M (SE)	34.5 (1.4)	73.7	46.8
Sex	Girls, M (SE)	36.5 (1.9)	69.4	52.6
	Boys, M (SE)	32.2 (2.0)	78.6	41.0
Age [Years]	10, M (SE)	41.4 (3.4)	63.4	65.3
	11, M (SE)	25.0 (2.5)	66.1	37.8
	12, M (SE)	29.7 (3.3)	70.5	42.1
	13, M (SE)	33.3 (4.3)	75.6	44.0
	14, M (SE)	38.7 (3.6)	77.9	49.7
	15, M (SE)	35.4 (2.5)	81.0	43.7
BMI	Underweight, M (SE)	30.3 (4.3)	81.1	37.4
	Normal body weight, M (SE)	34.1 (1.6)	72.8	46.8
	Overweight, M (SE)	38.5 (3.9)	72.8	52.9
	Obesity, M (SE)	41.6 (4.1)	70.6	58.9

M—mean; SE—standard error of the mean.

**Table 7:** Mean fat intake (g/day) by girls with age and nutritional status. Percentage of recommended fat intake according to the weighted dietary reference intake.

Age [Years]		Fat	Weighted Dietary Reference Intake	% of Intake Recommendation
	Total. M (SE)	36.5 (1.9)	69.4	52.6
10		39.8 (5.4)	60.8	65.4
	Underweight. M (SE)	-	-	-
	Normal weight. M (SE)	43.1 (5.4) ^a^	60.8	70.9
	Overweight. M (SE)	19.3 (7.4) ^a^	60.8	31.7
	Obesity. M (SE)	-	-	-
11		27.5 (3.9)	63.8	43.1
	Underweight. M (SE)	-	-	-
	Normal weight. M (SE)	25.7 (3.9) ^a^	63.8	40.3
	Overweight. M (SE)	51.3 (0.1) ^b^	63.8	80.4
	Obesity. M (SE)	-	-	-
12		29.5 (3.9)	67.4	43.7
	Underweight. M (SE)	-	-	-
	Normal weight. M (SE)	29.5 (3.9)	67.4	43.7
	Overweight. M (SE)	-	-	-
	Obesity. M (SE)	-	-	-
13		29.4 (4.3)	70.4	41.8
	Underweight. M (SE)	18.8 (0.1) ^a^	70.4	26.7
	Normal weight. M (SE)	27.9 (4.3) ^a^	70.4	39.6
	Overweight. M (SE)	35.8 (6.3) ^a^	70.4	50.9
	Obesity. M (SE)	-	-	-
14		46.3 (4.4)	72.6	63.8
	Underweight. M (SE)	-	-	-
	Normal weight. M (SE)	46.3 (4.4)	72.6	63.8
	Overweight. M (SE)	-	-	-
	Obesity. M (SE)	-	-	-
15		37.2 (2.5)	74.0	50.3
	Underweight. M (SE)	-	-	-
	Normal weight. M (SE)	35.9 (4.8) ^a^	74.0	48.5
	Overweight. M (SE)	40.2 (3.5) ^a^	74.0	54.3
	Obesity. M (SE)	35.5 (5.5) ^a^	74.0	50.0

M—mean, SE—standard error of the mean. a, b—statistically significant differences at *p* < 0.05; a, a—no statistically significant differences at *p* < 0.05.

**Table 8:** Mean fat intake (g/day) by boys with age and nutritional status. Percentage of recommended fat intake according to the weighted dietary reference intake.

Age [Years]		Fat	Weighted Dietary Reference Intake	% of Intake Recommendation
	Total. M (SE)	32.2 (2.0)	78.6	41.0
10		42.5 (3.4)	65.5	64.9
	Underweight. M (SE)	-	-	-
	Normal weight. M (SE)	30.6 (3.4) ^a^	65.5	46.7
	Overweight. M (SE)	73.1 (0.1)	65.5	111.6
	Obesity. M (SE)	50.7 (0.0) ^b^	65.5	77.4
11		21.7 (2.5)	69.1	31.4
	Underweight. M (SE)	9.4 (0.2) ^a^	69.1	13.6
	Normal weight. M (SE)	24.5 (3.9) ^a^	69.1	35.5
	Overweight. M (SE)	21.9 (1.6) ^a^	69.1	31.7
	Obesity. M (SE)	-		
12		29.9 (3.4)	73.9	40.5
	Underweight. M (SE)	38.2 (7.5) ^b^	73.9	51.7
	Normal weight. M (SE)	44.5 (6.9) ^b^	73.9	60.2
	Overweight. M (SE)	11.8 (0.5) ^a^	73.9	16.0
	Obesity. M (SE)	-		
13		36.1 (4.8)	79.5	45.4
	Underweight. M (SE)	32.7 (8.4) ^a^	79.5	41.1
	Normal weight. M (SE)	26.5 (5.9) ^a^	79.5	33.3
	Overweight. M (SE)	94.2 (0.0) ^b^	79.5	118.5
	Obesity. M (SE)	-	-	-
14		28.1 (3.6)	85.3	32.9
	Underweight. M (SE)	20.8 (7.2) ^a^	85.3	24.4
	Normal weight. M (SE)	29.9 (2.3)	85.3	35.0
	Overweight. M (SE)	36.9 (10.2) ^a^	85.3	43.3
	Obesity. M (SE)	-	-	-
15		33.0 (3.5)	90.4	36.5
	Underweight. M (SE)	20.8 (10.7) ^a^	90.4	23.0
	Normal weight. M (SE)	29.9 (7.2) ^a^	90.4	33.1
	Overweight. M (SE)	39.6 (10.6) ^b^	90.4	43.8
	Obesity. M (SE)	-	-	-

M—mean, SE—standard error of the mean. a, b—statistically significant differences at *p* < 0.05; a, a—no statistically significant differences at *p* < 0.05.

**Table 9:** Average intake of saturated fatty acids (SFAs, g/day) by schoolchildren by sex, age, and nutritional status, % of SFA intake according to weighted average maximum allowable intake for a heterogeneous group of consumers.

		SFAs [g]	Weighted Average Maximum Allowed of SFAs [g]	% of Standard Coverage of SFAs
	Total, M (SE)	15.3 (0.6)	25.9	59.1
Gender	Girls, M (SE)	16.1 (0.9)	24.4	66.0
	Boys, M (SE)	14.4 (0.9)	27.6	52.2
Age [Years]	10, M (SE)	18.9 (1.8)	22.3	84.8
	11, M (SE)	10.8 (1.0)	23.2	46.6
	12, M (SE)	12.7 (1.5)	24.7	51.4
	13, M (SE)	14.3 (1.9)	26.5	54.0
	14, M (SE)	17.2 (1.6)	27.3	63.0
	15, M (SE)	16.0 (1.2)	28.4	56.3
BMI	Underweight, M (SE)	13.8 (2.0)	28.4	48.6
	Normal body weight, M (SE)	14.8 (0.7)	25.5	58.0
	Overweight, M (SE)	18.0 (1.9)	25.5	70.6
	Obesity, M (SE)	18.3 (1.6)	24.8	73.8

**Table 10:** Average intake of saturated fatty acids (SFA g/day) by girls, taking into account age and nutritional status. Percentage of SFA intake according to the weighted average maximum allowable intake for a heterogeneous group of consumers.

Age [Years]		SFA [g]	Weighted Maximum Allowable SFA Intake [g]	% of Allowable SFA Intake
	Total. M (SE)	16.1 (0.9)	24.4	66.0
10		18.1 (2.7)	21.3	85.0
	Underweight. M (SE)	-	-	-
	Normal weight. M (SE)	19.7 (2.8) ^a^	21.3	92.5
	Overweight. M (SE)	8.4 (7.7) ^a^	21.3	39.4
	Obesity. M (SE)	-	-	-
11		11.3 (1.5)	22.4	50.4
	Underweight. M (SE)	-	-	-
	Normal weight. M (SE)	10.6 (1.5) ^a^	22.4	47.3
	Overweight. M (SE)	20.4 (0.2) ^a^	22.4	91.1
	Obesity. M (SE)	-	-	-
12		12.5 (1.9)	23.6	53.0
	Underweight. M (SE)	-	-	-
	Normal weight. M (SE)	12.5 (1.9)	23.6	53.0
	Overweight. M (SE)	-	-	-
	Obesity. M (SE)	-	-	-
13		13.8 (3.2)	24.7	55.9
	Underweight. M (SE)	8.8 (0.1) ^a^	24.7	35.6
	Normal weight. M (SE)	12.6 (3.5) ^a^	24.7	51.0
	Overweight. M (SE)	17.8 (8.2) ^a^	24.7	72.1
	Obesity. M (SE)	-	-	-
14		20.3 (1.9)	25.5	79.6
	Underweight. M (SE)	-	-	-
	Normal weight. M (SE)	20.3 (1.9)	25.5	79.6
	Overweight. M (SE)	-	-	-
	Obesity. M (SE)	-	-	-
15.		16.8 (1.6)	26.0	64.6
	Underweight. M (SE)	-	-	-
	Normal weight. M (SE)	15.7 (2.2) ^a^	26.0	60.4
	Overweight. M (SE)	19.1 (3.1) ^a^	26.0	73.5
	Obesity. M (SE)	15.8 (2.1) ^a^	26.0	60.8

M—mean, SE—standard error of the mean. a, a—no statistically significant differences at *p* < 0.05.

**Table 11:** Average intake of saturated fatty acids (SFA g/day) by boys, taking into account age and nutritional status. Percentage of SFA intake according to the weighted average maximum allowable intake for a heterogeneous group of consumers.

Age [Years]		SFA [g]	Weighted Maximum Allowable SFA Intake [g]	% of Allowable SFA Intake
	Total. M (SE)			
10		19.4 (2.4)	23.0	84.3
	Underweight. M (SE)	-	-	-
	Normal weight. M (SE)	13.3 (1.8) ^a^	23.0	57.8
	Overweight. M (SE)	36.2 (5.8)	23.0	157.4
	Obesity. M (SE)	21.9 (0.1) ^b^	23.0	95.2
11		10.2 (1.0)	24.3	42.0
	Underweight. M (SE)	4.6 (0.1) ^a^	24.3	18.9
	Normal weight. M (SE)	10.9 (1.2) ^a^	24.3	44.9
	Overweight. M (SE)	11.7 (1.5) ^a^	24.3	48.1
	Obesity. M (SE)	-	-	-
12		12.9 (2.3)	25.9	49.8
	Underweight. M (SE)	16.5 (4.5) ^b^	25.9	63.7
	Normal weight. M (SE)	18.8 (5.0) ^b^	25.9	72.6
	Overweight. M (SE)	5.4 (0.3) ^a^	25.9	20.8
	Obesity. M (SE)	-	-	-
13		14.8 (2.4)	27.9	53.0
	Underweight. M (SE)	14.0 (3.9) ^a^	27.9	50.2
	Normal weight. M (SE)	10.0 (6.5) ^a^	27.9	35.8
	Overweight. M (SE)	40.6 (0.1) ^b^	27.9	145.5
	Obesity. M (SE)	-	-	-
14		12.8 (2.5)	29.9	42.8
	Underweight. M (SE)	10.0 (5.2) ^a^	29.9	33.4
	Normal weight. M (SE)	13.6 (3.3) ^a^	29.9	45.5
	Overweight. M (SE)	15.6 (6.3) ^a^	29.9	52.2
	Obesity. M (SE)	-	-	-
15		15.0 (1.7)	31.7	47.3
	Underweight. M (SE)	18.5 (3.2) ^a^	31.7	58.4
	Normal weight. M (SE)	13.6 (3.3) ^a^	31.7	42.9
	Overweight. M (SE)	13.7 (0.1) ^a^	31.7	43.2
	Obesity. M (SE)	-	-	-

M—mean, SE—standard error of the mean. a, b—statistically significant differences at *p* < 0.05; a, a—no statistically significant differences at *p* < 0.05.

**Table 12:** Average intake of trans fatty acids isomers (TFAs, g/day) by schoolchildren by gender, age, and nutritional status, % TFAs intake according to weighted average maximum allowable intake for a heterogeneous group of consumers.

		TFAs [g]	Weighted Average Maximum Allowed of TFAs [g]	% of Standard Coverage of TFAs
	Total, M (SE)	2.5 (0.1)	2.6	96.2
Gender	Girls, M (SE)	2.5 (0.2)	2.4	104.2
	Boys, M (SE)	2.4 (0.2)	2.8	85.7
Age [years]	10, M (SE)	3.6 (0.2)	2.2	163.6
	11, M (SE)	1.5 (0.3)	2.3	65.2
	12, M (SE)	1.8 (0.3)	2.5	72.0
	13, M (SE)	2.2 (0.5)	2.7	81.5
	14, M (SE)	2.7 (0.4)	2.7	100.0
	15, M (SE)	2.8 (0.3)	2.8	100.0
BMI	Underweight, M (SE)	2.4 (0.5)	2.8	85.7
	Normal body weight, M (SE)	2.3 (0.2)	2.6	88.5
	Overweight, M (SE)	2.9 (0.4)	2.6	111.5
	Obesity, M (SE)	3.2 (0.5)	2.5	128.0

**Table 13:** Mean intake of trans fatty acid isomers (TFA g/day) by girls by age and nutritional status. Percentage of TFA intake by weighted average maximum allowable intake for a heterogeneous group of consumers.

Age [Years]		TFA [g]	Weighted Maximum Allowable TFA Intake [g]	% of Allowable TFA Intake
	Total. M (SE)	2.5 (0.2)	2.4	104.2
10		3.6 (0.2)	2.1	171.4
	Underweight. M (SE)	-	-	-
	Normal weight. M (SE)	2.5 (0.4) ^a^	2.1	119.0
	Overweight. M (SE)	3.9 (1.1) ^a^	2.1	185.7
	Obesity. M (SE)	-	-	-
11		1.5 (0.3)	2.2	68.2
	Underweight. M (SE)	-	-	-
	Normal weight. M (SE)	1.5 (0.4) ^a^	2.2	68.2
	Overweight. M (SE)	2.1 (0.1) ^a^	2.2	95.5
	Obesity. M (SE)	-	-	-
12		1.8 (0.3)	2.5	88.0
	Underweight. M (SE)	-	-	-
	Normal weight. M (SE)	1.8 (0.3)	2.5	88.0
	Overweight. M (SE)	-	-	-
	Obesity. M (SE)	-	-	-
13		2.2 (0.5)	2.5	88.0
	Underweight. M (SE)	1.0 (0.1) ^a^	2.5	40.0
	Normal weight. M (SE)	1.7 (0.1) ^a^	2.5	68.0
	Overweight. M (SE)	2.9 (0.4) ^a^	2.5	116.0
	Obesity. M (SE)	-	-	-
14		2.7 (0.4)	2.5	108.0
	Underweight. M (SE)	-	-	-
	Normal weight. M (SE)	2.7 (0.4)	2.5	108.0
	Overweight. M (SE)	-	-	-
	Obesity. M (SE)	-	-	-
15		2.8 (0.3)	2.6	107.7
	Underweight. M (SE)	-	-	-
	Normal weight. M (SE)	2.6 (0.7) ^a^	2.6	100.0
	Overweight. M (SE)	3.7 (1.3) ^a^	2.6	142.0
	Obesity. M (SE)	1.7 (0.4) ^a^	2.6	65.4

M—mean, SE—standard error of the mean. a, a—no statistically significant differences at *p* < 0.05.

**Table 14:** Mean intake of trans fatty acid isomers (TFA g/day) by boys by age and nutritional status. Percentage of TFA intake by weighted average maximum allowable intake for a heterogeneous group of consumers.

Age [Years]		TFA [g]	Weighted Maximum Allowable TFA Intake [g]	% of Allowable TFA Intake
	Total. M (SE)			
10. M (SE)		2.8 (0.3)	2.3	121.7
	Underweight. M (SE)	-	-	-
	Normal weight. M (SE)	2.1 (0.3) ^a^	2.3	91.3
	Overweight. M (SE)	4.4 (0.5)	2.3	191.3
	Obesity. M (SE)	3.9 (0.0) ^b^	2.3	169.5
11		1.5 (0.3)	2.4	62.5
	Underweight. M (SE)	0.2 (0.0) ^a^	2.4	8.3
	Normal weight. M (SE)	1.8 (0.5) ^b^	2.4	75.0
	Overweight. M (SE)	1.6 (0.1) ^b^	2.4	66.6
	Obesity. M (SE)	-	-	-
12		2.1 (0.4)	2.6	80.8
	Underweight. M (SE)	2.4 (0.8) ^a^	2.6	92.3
	Normal weight. M (SE)	1.9 (0.5) ^a^	2.6	73.1
	Overweight. M (SE)	1.8 (0.3) ^a^	2.6	69.2
	Obesity. M (SE)	-	-	-
13		3.2 (0.8)	2.8	114.3
	Underweight. M (SE)	3.0 (1.7) ^a^	2.8	107.1
	Normal weight. M (SE)	1.5 (1.8) ^a^	2.8	53.6
	Overweight. M (SE)	12.8 (0.1) ^b^	2.8	457.1
	Obesity. M (SE)	-	-	-
14.		2.4 (0.7)	3.0	80.0
	Underweight. M (SE)	2.3 (1.2)	3.0	76.7
	Normal weight. M (SE)	2.7 (1.0)	3.0	90.0
	Overweight. M (SE)	1.4 (0.3)	3.0	46.7
	Obesity. M (SE)	-	-	-
15		2.3 (0.3)	3.2	71.9
	Underweight. M (SE)	2.2 (1.2) ^a^	3.2	68.8
	Normal weight. M (SE)	2.6 (1.0) ^a^	3.2	81.3
	Overweight. M (SE)	1.4 (0.4) ^a^	3.2	43.8
	Obesity. M (SE)	-	-	-

M—mean, SE—standard error of the mean. a, b—statistically significant differences at *p* < 0.05; a, a—no statistically significant differences at *p* < 0.05.

**Table 15:** Number and percentage of people exceeding fat, SFA, and TFA intake standards.

Total		Total	FAT		SFA		TFA	
		N	n	%	n	%	n	%
		362	44	12.2	72	19.9	115	31.8
Gender	Girls	192	28	14.6	45	23.4	59	30.7
	Boys	170	16	9.4	27	15.9	56	32.9
Age [years]	10	50	12	24.0	13	26.0	32	64.0
	11	49	2	4.1	4	8.2	9	14.3
	12	50	5	10.0	10	20.0	15	30.0
	13	47	9	19.1	9	19.1	11	23.4
	14	82	12	14.6	20	24.4	22	26.8
	15	84	4	4.8	16	19.0	26	33.3
BMI	Underweight	43	4	9.3	9	20.9	14	32.6
	Normal body weight	253	27	10.7	44	17.4	73	28.9
	Overweight	56	12	21.4	17	30.4	24	42.8
	Obesity	10	1	10.0	2	20.0	4	40.0

## Data Availability

The data presented in this study are available on request from the corresponding author due to privacy reasons.
